# Nasal Bridles for Securing Nasoenteric Feeding Tubes: A Review of Clinical Effectiveness and Potential Complications

**DOI:** 10.7759/cureus.8325

**Published:** 2020-05-28

**Authors:** Faisal Inayat, Asad Ur Rahman, Talal Almas, Effa Zahid, Xaralambos Zervos

**Affiliations:** 1 Internal Medicine, Allama Iqbal Medical College, Lahore, PAK; 2 Gastroenterology, Cleveland Clinic Florida, Weston, USA; 3 Internal Medicine, Royal College of Surgeons in Ireland, Dublin, IRL; 4 Internal Medicine, Services Institute of Medical Sciences, Lahore, PAK; 5 Transplant Hepatology, Cleveland Clinic Florida, Weston, USA

**Keywords:** enteral nutrition, nasoenteric tubes, nasal bridles, feeding tube dislodgment, magnet detachment, clinical effectiveness, procedural complications

## Abstract

Nasal bridle is a feeding tube retaining device that is now increasingly used worldwide. While common complications tend to be minor, it is important to remain vigilant for newer adverse events. We hereby delineate the case of an elderly female who required nasoenteric feeding tube following simultaneous liver-kidney transplantation. Nasal bridle placement was warranted owing to her significant frailty and poor mentation. Due to her extreme agitation during the procedure, bridle insertion could not be completed. Upon removal of the probe, unprompted detachment of the magnetic tip was noted. Radiological workup revealed the dislodged magnet in the sphenoid sinus. Subsequently, she underwent an uneventful endoscopic sinus surgery, resulting in successful retrieval of the magnet. This paper highlights the spontaneous magnet avulsion from a bridling system and serves the purpose of community awareness regarding this unusual procedural complication. Additionally, we aim to evaluate the efficacy of the nasal bridle, further accentuating its advantages and possible complications.

## Introduction

In 1980, McGuirt and Strout first described securing nasoenteric tubes by employing a nasal bridle [1]. Later on, Gunn et al. modified the retaining device by introducing a magnetic system along with 1/8-inch umbilical tape [2]. AMT Bridle™ system refers to the Applied Medical Technology that originated in Brecksville (OH, USA). Currently, the bridle is inserted using magnets attached to the distal ends of the catheter and probe. After inserting the catheter into one nostril and the probe into the other, the magnets come in contact posterior to the vomer bone, forming a loop or ‘’bridle’’ of tape around the bone. The ends of a feeding tube are securely connected with the tape. This device has been used to maintain the short-term nutrition supplementation in patients who are at risk for accidental dislodgement of the standard nasoenteric tubes.

Nasal bridle effectively reduces the tube dislodgement rates compared to traditional modalities used to this end [2]. Consequently, it helps to achieve quick recovery, decrease the duration of hospital stay, minimize the need for repeat imaging, and save time by reducing the frequency of nasoenteric tube replacement. However, the increased utility of bridling means that novel adverse events can possibly be encountered in clinical practice. In this study, we describe an unusual complication pertaining to spontaneous magnet detachment from the probe of a nasal bridle, requiring a subsequent sinus surgery for its removal. Clinical and ancillary staff should be aware of this potential problem due to the risk of aspiration of a dislodged magnet or its lodgment into unusual locations. Furthermore, we review the pertinent medical literature for clinical benefits and potential complications associated with the use of this device.

## Case presentation

**Figure 1:**
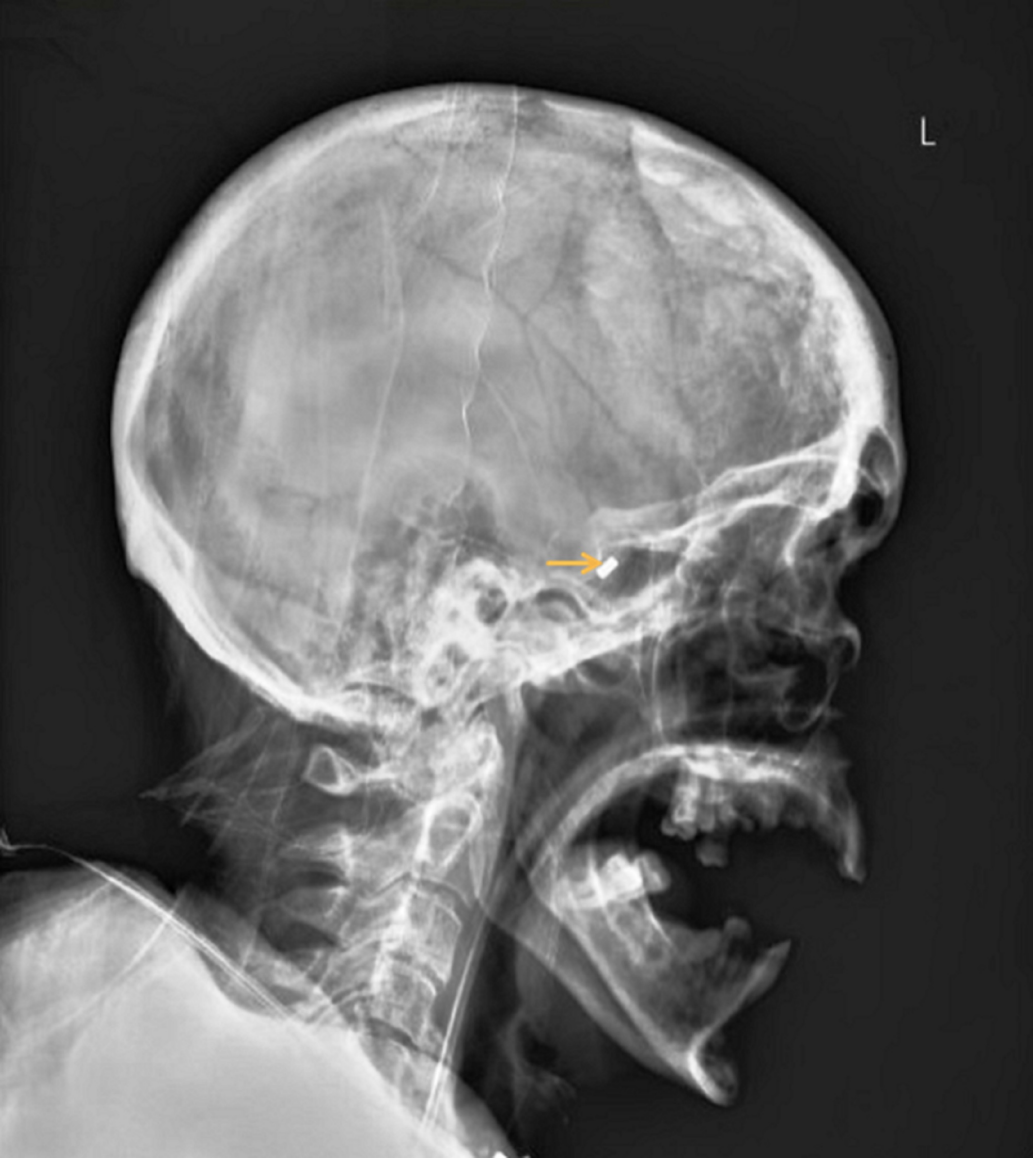
X-ray lateral view of paranasal sinuses showing a radiopaque object (arrow) lodged at the sphenoid os.

A 66-year-old female underwent an uneventful simultaneous liver-kidney transplantation 10 days prior to the current presentation. Her medical history included steatohepatitis-related cirrhosis and chronic renal insufficiency following diabetic nephropathy. Owing to her significant baseline frailty, the patient showed a prolonged postoperative recovery. She continued to have a poor appetite and fluctuating mentation after the surgery. A nasoenteric tube was thus placed through the left nostril to ameliorate the caloric deficit. After the procedure, magnetic nasal bridle (AMT Bridle, Applied Medical Technology, Brecksville, OH, USA) insertion was attempted. However, she became extremely agitated during the bridle placement and intermagnetic linkage could not be achieved. Upon removal of the probe, it was noted that the magnet was missing from its distal end. The otorhinolaryngology service was consulted. An urgent nasal endoscopy was performed, but the magnet could not be visualized. A plain radiograph of the paranasal sinuses showed a radiopaque object lodged at the sphenoid os (Figure 1).

**Figure 2:**
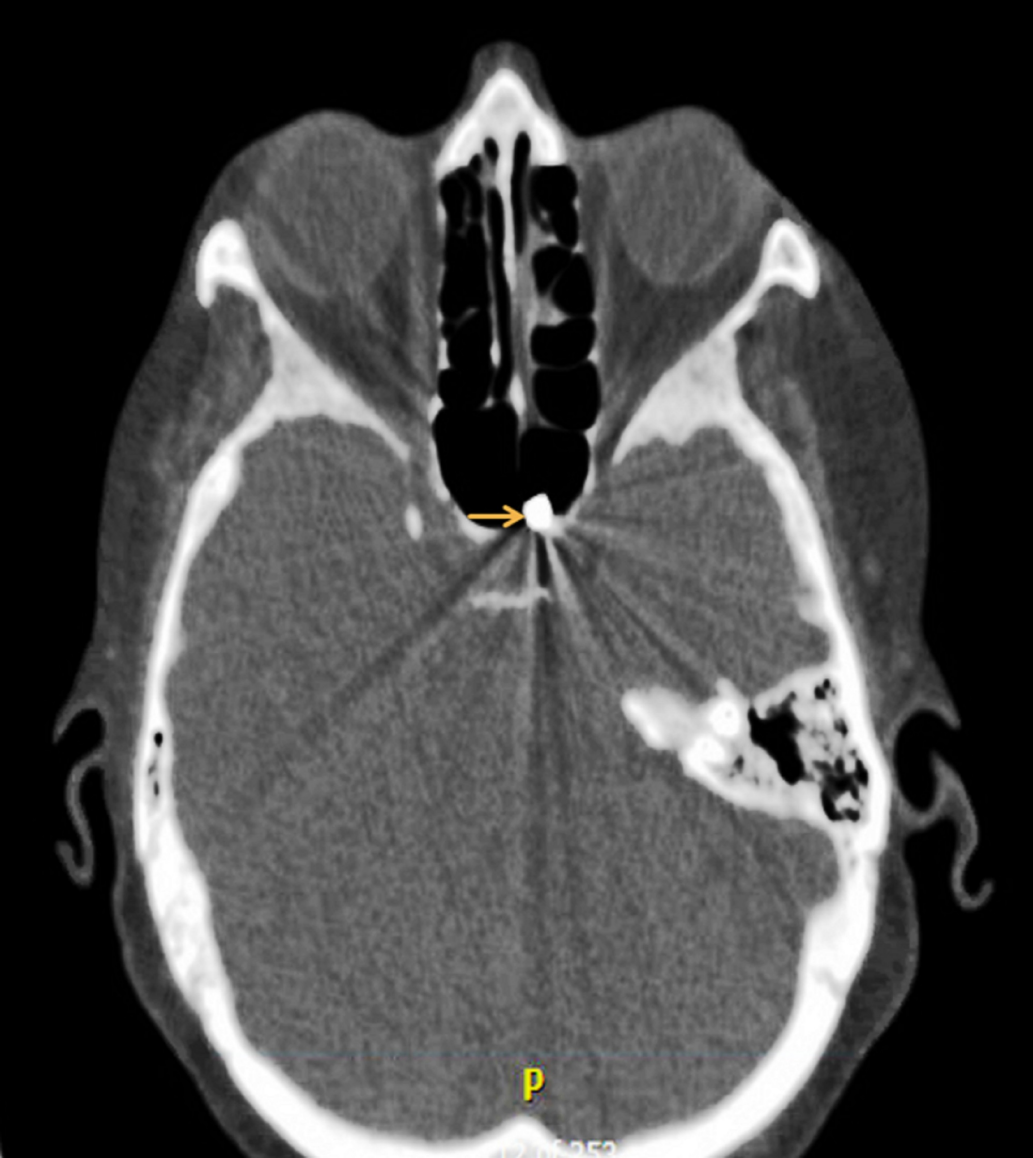
Computed tomography axial view at the level of paranasal sinuses showing the magnet with associated metallic artifact (arrow), originating from the left sphenoid sinus.

Computed tomography (CT) of the head and neck confirmed the magnet with the associated metallic artifact, originating from the sphenoid sinus (Figure 2).

**Figure 3:**
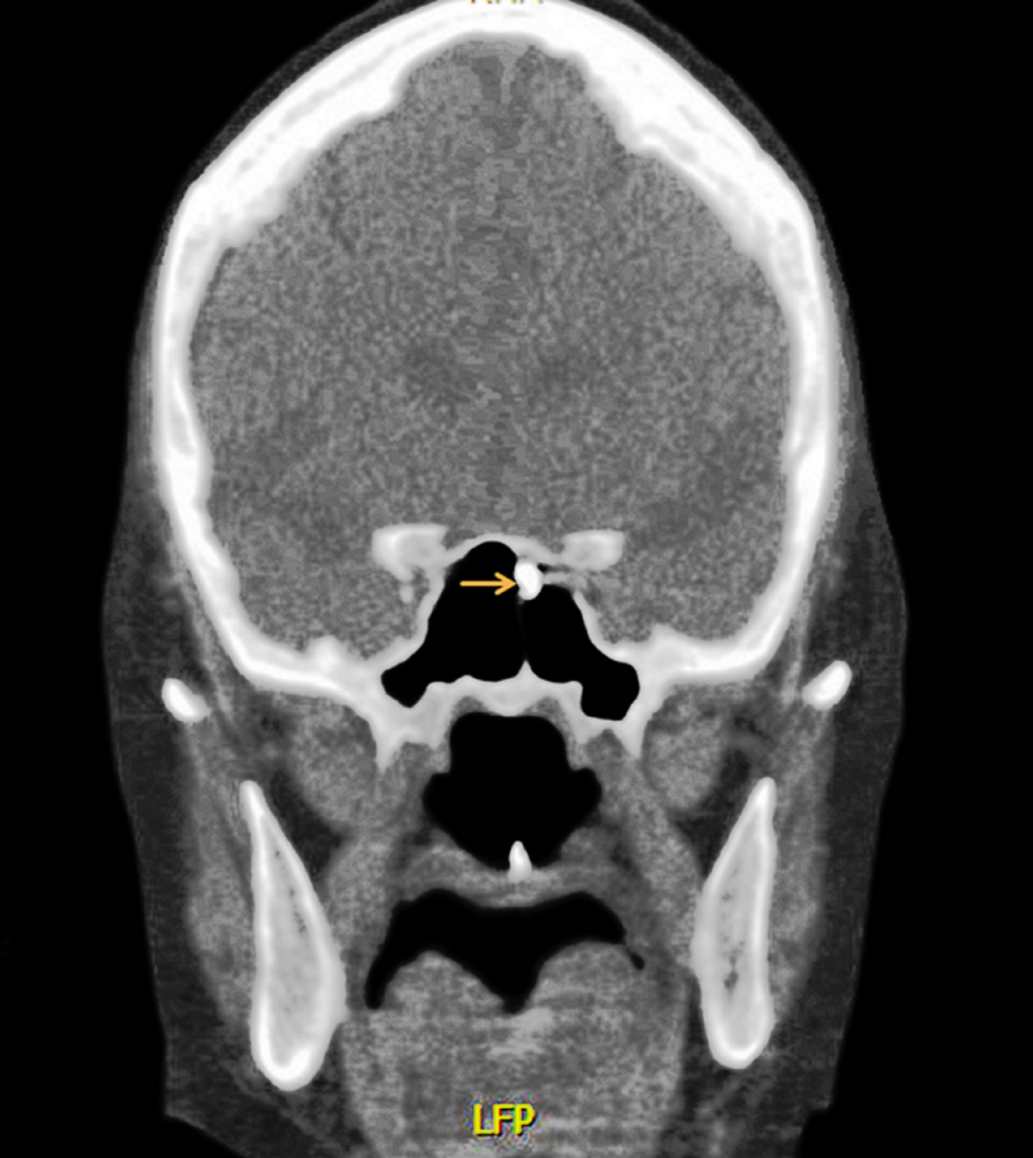
Computed tomography coronal view through the sphenoid sinus showing an oval, well-circumscribed, hyperdense opacity (arrow) in the superomedial aspect of left sphenoid sinus, consistent with the dropped magnet.

The artifact appeared as small, well-circumscribed, hyperdense opacity in the superomedial aspect of the left sphenoid sinus (Figure 3).

A multidisciplinary team planned a CT-guided endoscopic sinus surgery. The surgical intervention resulted in the successful retrieval of the magnet. Thereafter, she completely recovered without the need for a percutaneous gastrostomy tube (PEG) placement.

## Discussion

Enteral nutrition plays a vital role in the nourishment of patients who are unable to maintain their volitional intake. This technique ensures the appropriate supplementation of required nutrients, which in turn hastens the recovery in such patients [3]. Due to their frequent placement in critically ill patients, accidental dislodgment of feeding tubes may pose a clinical dilemma. Therefore, the use of retaining devices for the securement of nasoenteric tubes has become imperative. As complications abound, hospitals have formulated clinical protocols that govern the placement of nasal bridles. These protocols primarily outline the indications for bridle placement, such as the frequency of feeding tube dislodgement, further positing that bridle placement must be considered in patients who have pulled out the nasoenteric tube at least three times within one week. These guidelines, as they pertain to patients with stroke, have also been approved by the Royal College of Physicians in the United Kingdom [4]. Although a multitude of studies have vouched for the safety and efficacy of nasal bridles in the United States, the specific guidelines mandating their use remain elusive.

The data regarding the use of nasal bridles show that it effectively decreases the feeding tube dislodgement rate [5,6]. Therefore, the need for tube replacement decreases, thereby reducing the inveterate stresses of tubal re-insertion. Bechtold et al. demonstrated in their meta-analysis that tubal dislodgement occurred in 40% of the patients in whom conventional securing methods were used compared to merely 14% of those in the nasal bridle group. It is imperative to highlight that these results were statistically significant (odds ratio [OR], 0.16; 95% confidence interval [CI], 0.10-0.27; P<0.01) [7]. Parks et al. divulged that securing the feeding tube with nasal bridles in burn patients reduced the average number of tubal replacements required per day when compared to traditional securing modalities (0.26 vs 0.44; P<0.05) [8]. It further purported the notion that bridles help to achieve decreased dislodgement rates. Pain and discomfort are important factors that warrant consideration during bridle insertion. Beaven et al. delineated that the bridle-associated pain was noted in 28% and 41% in the bridle and control groups, respectively. Therefore, contrary to the prevalent belief, it is noteworthy that nasal bridles do not elicit increased pain levels [9]. Additionally, Al-Khudari et al. noted that pain associated with bridling was significantly decreased nine days after the initial administration [10]. With the provision of sustained nasoenteric nutrition, nasal bridles can significantly improve patient recovery rates. Prior research has convincingly demonstrated that nasal bridles expedite the recovery and help early initiation of swallowing, consequently expunging the need for PEG placement [11,12].

While the efficacy of nasal bridle in yielding ameliorated nasoenteric feeding outcomes is indubitable, perceptions about associated complications can limit its uptake. Due to the fact that a probe is initially required, the bridling procedure can elicit minor complications such as epistaxis and nasal ulceration [13,14]. Initial insertional procedures might also mean that complications such as kinking and cracking of the nasoenteric tube might arise from the bridle placement. A meta-analysis further found that the risk of cutaneous complications was increased in the bridle group (13%) when compared to the group with conventional securing modalities (3%) [15]. No serious complications were observed with the use of nasal bridles, further elaborating their efficacy and safety profile. Furthermore, the incidence of sinusitis was decreased in the nasal bridle group [15]. Thus, most complications associated with the use of nasal bridle have been mild that can be easily managed. At the time of bridle placement, closely following the manufacturer’s manual is likely to help evade these reported problems. In order to further decrease the rate of minor complications, a systems-based approach should be applied during bridle placement.

Recently, several newer adverse events of bridling have been documented. Saunders and Osborne reported a rare case of flexible guidewire retention, which was initially used to insert a nasal bridle [16]. The guidewire had been retained in the left nasal cavity and was discovered upon an anterior rhinoscopy, 12 months after bridle insertion. While no debilitating sequelae were evident, this case alludes to the unmet need to develop the correct insertion and withdrawal guidelines for nasal bridles [16]. Similarly, a report by Jackson and Sharma described two cases of the retainment of the insertional stylet. Although these dislodged stylets were removed promptly at the bedside, they nevertheless point towards the riveting possibility of stylet retainment after bridling [17]. The spontaneous magnet avulsion, as in this case, remains a remarkably rare occurrence. Smith et al. elucidated a case of magnetic tip detachment from a nasal bridle in the United Kingdom [18]. During the process of bridle insertion, the magnet detached and fell into the left sphenoid sinus. The artifact was eventually discovered upon a CT scan [18]. Additionally, Puricelli et al. have reported the first case of an avulsed bridle magnet in the United States, arguing that although a rare complication, magnetic tip dislodgement can elicit debilitating consequences for the patient [19]. Therefore, to our knowledge, the present report represents only the second case of magnetic dislodgement from a nasal bridle in the United States. These newer procedural complications invariably warrant additional vigilance with the use of bridle system. 

The minor complications associated with bridling can be circumvented with proper insertional technique. In order to reduce the cutaneous complications, the external placement of the bridle should be evaluated and finalized in such a way that it does not exert undue traction on the nose of adjacent structures. A standard-check procedure should be adhered to upon the initial insertion of the bridle, ensuring that the guidewire is safely retracted after the administration of the bridle so that no component is left in situ. In terms of dealing with these rarer complications, such as magnet detachment, the bridle system should be assembled in accordance with the manufacturer’s guidelines. A multitude of manufacturers produce these bridles, making it important to adhere to the instructions detailed in the respective manual. Furthermore, the clinical staff should ensure that the magnet is securely fitted into the bridle prior to insertion. Although it is generally believed that the magnet is well embedded into the bridle, it can sometimes come loose. Therefore, pre-procedural checks can help avert this unusual complication. We also recommend bridle placement prior to nasoenteric tube insertion as this will leave adequate space in the nasal cavity for catheter passage. This sequence has also been authorized by the bridle manufacturer’s insertion guidelines. It is expected that patients may still develop such problems in clinical practice. Therefore, we emphasize that magnetic resonance imaging should not be performed as a part of the radiological workup in patients with suspected bridle magnetic avulsion.

Additional measures might also help in evading the procedural complications of bridle placement. Physicians and ancillary staff must ensure that they have thoroughly perused the training manuals and that at the time of insertion, they closely follow a standard checklist. Certain anatomical observations might also contribute towards the uneventful bridle insertion. Puricelli et al. posited that insertion of the bridle at the level of the nasal floor is likely to proceed unhindered, and it is pivotal to ensure that the bridle is parallel to the nasal floor upon insertion [19]. Furthermore, the pre-insertional application of a nasal decongestant is likely to diminish the turbinate dimensions, enabling smoother passage. This may also help to circumscribe the incidence of minor vessel-related complications, such as epistaxis. Finally, local analgesics might be applied to reduce patient discomfort. However, it should mostly be reserved for instances in which exceptionally poor bridle tolerance is observed.

## Conclusions

Nasal bridles help to provide sustained enteral nutrition in patients incapable of maintaining volitional feeding. The benefits afforded by this device significantly outweigh the associated minor complications. Therefore, this technique is now increasingly being used for optimal enteral feeding across the United States. This study presents an important clinical update regarding unusual adverse events associated with bridle insertion. Physicians should be aware of procedural complications like spontaneous magnet detachment due to the risk of aspiration or its lodgment into unusual locations, potentially culminating in a devastating outcome or unnecessary surgical intervention.
